# Advanced Porous Nanomaterials: Synthesis, Properties, and Applications

**DOI:** 10.3390/nano14191602

**Published:** 2024-10-03

**Authors:** Yannick Guari

**Affiliations:** ICGM, Université Montpellier, CNRS, ENSCM, 34095 Montpellier, France; yannick.guari@umontpellier.fr

Porous nanomaterials have emerged as one of the most versatile and valuable classes of materials, captivating the attention of both scientists and engineers due to their exceptional functional and structural properties. Characterized by pore sizes less than 100 nm, these materials exhibit a remarkable combination of high surface area, low bulk density, thermal stability, and tunable permeability. This makes them indispensable in a wide range of applications, including catalysis, drug delivery, gas separation, energy storage, and beyond. Their unique properties result from their ability to interact with molecules and ions in confined environments, which can be tailored through careful synthesis and functionalization processes. The rapid development of advanced porous nanomaterials continues to drive breakthroughs in various fields, particularly in energy management, environmental sustainability, and biomedicine.

This Special Issue focuses on recent advancements in the synthesis, characterization, and application of various types of porous nanomaterials, with a particular emphasis on silica, carbon-based materials, metal–organic frameworks (MOFs), and zeolites. The collection of ten publications featured here demonstrates the breadth of current research efforts aimed at optimizing the performance of these materials for specific applications, from improving semiconductor polishing to enhancing the removal of contaminants in environmental settings (Table 1).

**Silica Nanoparticles**
  *Enhancing Slurry Stability and Surface Flatness of Silicon Wafers through Organic Amine-Catalyzed Synthesis Silica Sol (Contribution 1) [1]*

The demand for high-performance semiconductor devices has increased with advancements in AI, cloud computing, and IoT, necessitating high-quality silicon wafers [2]. In wafer production, the grinding and polishing stages are vital for surface smoothness, enhancing the final product’s performance [3]. Colloidal silica nanoparticles, often synthesized using the Stöber process [4,5,6,7,8], are preferred for chemical mechanical polishing (CMP) due to their stability and moderate hardness [9]. Organic amines, such as ethanolamine, have been shown to increase the polishing rate while reducing surface roughness and eliminating metal contamination risks [10]. However, amines can destabilize silica sol under certain conditions. This study addresses the challenges of slurry stability in chemical mechanical polishing (CMP) applications, a critical process in semiconductor manufacturing. The authors explore the use of organic amines as catalysts for synthesizing alkaline silica sols, which show improved stability and performance in silicon wafer polishing. By enhancing the surface flatness and reducing corrosion, this work contributes to advancements in CMP technology, demonstrating how tailored synthesis approaches can optimize material properties for industrial applications.

*Protocrystallinity of Monodispersed Ultra-Small Templated Mesoporous Silica Nanoparticles (Contribution 2) [11]*

Research on controlling the size of porous nanoparticles under 100 nm is crucial for applications in medical imaging, drug delivery, and catalysis. These nanosized particles offer advantages like improved diffusion control in living systems and the industrial design of adsorbents and catalysts [12,13,14,15]. Inorganic nanocargos, especially those that are silica-based, are valued for their porosity and potential for surface functionalization [16,17]. Mesoporous silica nanoparticles with well-ordered structures are typically over 50–70 nm [18]. Size control methods involve supersaturation, chemical quenching, and the use of additives [19,20,21]. These techniques help to stabilize small particles, though achieving well-ordered structures below 50 nm remains challenging. This work presents a novel synthesis approach for ultra-small mesoporous silica nanoparticles (US-MSNs), with the goal of achieving a protocrystalline state—a transitional form between amorphous and crystalline structures. These nanoparticles exhibit hierarchical porosity and unique packing properties, making them highly suitable for applications in separation and catalysis. The meticulous control over particle size and morphology highlights the importance of precision in nanoparticle synthesis when trying to achieve desired functionalities.

*Upscale Synthesis of Magnetic Mesoporous Silica Nanoparticles and Application to Metal Ion Separation: Nanosafety Evaluation (Contribution 3) [22]*

Core–shell magnetic mesoporous silica nanoparticles (MMSNs) have gained significant attention over the past decade due to their wide range of applications. They are used in cancer theranostics for magnetic resonance imaging and drug delivery [23,24], as sensors for detecting substances [25], and for extracting heavy metals and rare earth elements [26]. MMSNs have also shown potential in removing iron from biological media [27]. The first MMSNs were synthesized in 2008 by encapsulating iron oxide cores within mesoporous silica shells [28,29]. However, their synthesis remains complex, especially when scaling up, as it can lead to inconsistent results like multiple cores or incomplete structures. Scaling up the synthesis of MMSNs while maintaining structural integrity is a key challenge addressed in this paper. The authors successfully scale the production to the gram level and demonstrate the potential of functionalized MMSNs for metal ion separation. The study also emphasizes the importance of evaluating the nanosafety of these materials, confirming their low toxicity across various biological models; this is crucial for their potential use in environmental and biomedical applications.

**Metal–Organic Frameworks (MOFs)**
  *Sodium Alginate/UiO-66-NH_2_ Nanocomposite for Phosphate Removal (Contribution 4) [30]*

Phosphorus pollution from pesticides, detergents, and fertilizers is worsening, leading to excessive phosphates in water bodies and causing eutrophication, harmful algae growth, and ecological imbalance [31]. To address this, methods like precipitation, adsorption, and biological processes are used to remove phosphates from water [32,33,34,35,36]. Adsorption is particularly valuable, allowing phosphorus recovery for reuse in agriculture [37]. MOFs, with their high surface area, stability, and functional groups, have shown promise for phosphate adsorption [38,39,40,41,42,43]. However, issues like particle agglomeration and recycling challenges persist [44]. Sodium alginate (SA) offers a potential solution, forming stable hydrogels to enhance MOF performance [45,46,47]. The environmental issue of phosphate pollution is tackled using a modified MOF-based composite. The incorporation of UiO-66-NH2 within alginate microspheres enhances the material’s ability to adsorb phosphates from water, making it a promising candidate for water purification. The study provides a comprehensive assessment of the composite’s performance under different conditions, demonstrating its effectiveness in real-world applications, particularly in the context of wastewater treatment.

*The Complexity of Comparative Adsorption of C6 Hydrocarbons (Benzene, Cyclohexane, n-Hexane) at Metal–Organic Frameworks (Contribution 5) [48]*

Toxic volatile organic compounds (VOCs), including hydrocarbons, pose environmental concerns, especially in industrial processes [49]. Traditional VOC removal methods use activated carbon or zeolites, but they lack selectivity for similar VOCs [50]. MOFs offer a more adaptable solution due to their tunable pore sizes and functional groups, allowing for selective adsorption of VOCs [51,52,53]. MOFs have been shown to effectively adsorb VOCs like benzene, toluene, and hexane [54,55,56]. Their flexibility and defects can enhance selectivity and adsorption capacity [57,58]. However, while adsorption capacity has been well studied, MOF stability during long-term VOC exposure needs further investigation. This article delves into the complexity of hydrocarbon adsorption on various MOFs, highlighting how pore structure and functional groups influence adsorption behavior. The study provides insights into the selective adsorption of different hydrocarbons, which is vital for applications such as gas separation and purification. The intricate interplay of pore size, ligand effects, and electrostatics underscores the challenges and opportunities in designing MOFs for targeted applications.

*Computational Screening of Metal–Organic Frameworks for Ethylene Purification from Ethane/Ethylene/Acetylene Mixture (Contribution 6) [59]*

Ethylene (C_2_H_4_) is a key chemical in the petroleum industry [60], typically separated from acetylene (C_2_H_2_) and ethane (C_2_H_6_) via energy-intensive methods like catalytic hydrogenation and cryogenic distillation [61]. MOFs offer a promising alternative for more energy-efficient separations due to their tunable pores and high surface areas. They show selectivity in separating C_2_H_2_/C_2_H_4_ [62,63,64] and C_2_H_4_/C_2_H_6_ [65,66,67,68,69] through size sieving, open metal sites, and specific interactions. While binary separations have been studied extensively, direct purification of the ternary C2 mixture is less explored. Recent studies highlight MOFs capable of separating all three gases simultaneously, offering a streamlined solution [70,71]. A computational approach is used to screen MOFs for the selective adsorption of ethylene from a ternary gas mixture. This work is significant for the chemical industry, where ethylene purification is a crucial step in many processes. By identifying high-performing MOFs through simulation, the study offers valuable guidelines for the design and optimization of adsorbent materials for gas separation, paving the way for more energy-efficient processes.

**Zeolites**
  *Effect of Nanoporous Molecular Sieves TS-1 on Electrical Properties of Crosslinked Polyethylene Nanocomposites (Contribution 7) [72]*

High-voltage direct current (HVDC) cables rely on crosslinked polyethylene (XLPE) for insulation due to its excellent electrical and thermal properties [73]. However, prolonged exposure to high-voltage DC fields causes space charge accumulation, leading to localized heating, discharges, and potential insulation breakdown [74,75,76,77]. Introducing fillers into the XLPE matrix, such as nanofillers and molecular sieves, helps improve the breakdown field strength and inhibit space charge buildup. Recent studies show that incorporating nano-structured fillers with specific pore sizes can limit electron impact ionization, improving the insulation’s strength and durability [77,78,79,80]. This approach is based on the nano-air gap effect, preventing traditional avalanche breakdown and enhancing HVDC cable performance. Zeolites are utilized here to enhance the electrical properties of XLPE nanocomposites, which are important for HVDC systems. The introduction of TS-1 zeolites improves the dielectric constants and breakdown field strength of the composites, highlighting the potential of nanoporous zeolites in enhancing the performance of insulating materials in energy transmission systems. This work showcases how the pore structure of zeolites can be leveraged to tailor material properties for specific electrical applications.

*Evaluation of the Hydrophilic/Hydrophobic Balance of 13X Zeolite by Adsorption of Water, Methanol, and Cyclohexane as Pure Vapors or as Mixtures (Contribution 8) [81]*

In gas separation and catalysis, water significantly impacts the efficiency of porous materials like activated carbons, zeolites, and MOFs by competing for active sites, particularly hindering CO_2_ and VOC capture [82,83,84]. The pre-removal of water in industrial applications adds cost and complexity. Among the studied materials, 13X FAU zeolite stands out as a promising benchmark due to its unique pore structure, which includes cages accessible to small molecules like water [85,86]. Its hydrophilic properties, coupled with its high adsorption capacity, make 13X FAU interesting for gas separations in humid conditions, where maintaining efficiency is crucial. The study explores the adsorption behavior of 13X zeolite with respect to different vapors, providing a detailed analysis of its hydrophilic/hydrophobic balance. This balance is critical for applications such as separation processes, where selectivity is paramount. The authors successfully simulate the adsorption isotherms, contributing to the fundamental understanding of zeolite behavior in mixed-vapor environments; this is essential for optimizing their performance in industrial separations.

**Carbon-Based Nanomaterials**
  *The Influences of Pore Blockage by Natural Organic Matter and Pore Dimension Tuning on Pharmaceutical Adsorption onto GO-Fe_3_O_4_ (Contribution 9) [87]*

Pharmaceuticals are emerging contaminants that, due to their widespread use and persistence in the environment, pose risks to public health and aquatic ecosystems [88]. While some are biodegradable, their increasing presence leads to environmental pseudo-persistence. For instance, Metformin (MET) and Diclofenac (DCF) are prevalent in water sources, with MET detected widely in wastewater and DCF affecting aquatic life even at trace levels [89,90,91]. Propranolol, used for hypertension, is also commonly found in rivers and estuaries [92,93,94]. When combining Graphene oxide (GO) with magnetite to form GO-Fe_3_O_4_, its effectiveness in removing pharmaceuticals from water improves, with the added benefit of magnetic recovery [95,96]. However, natural organic matter (NOM), including humic and fulvic acids, in wastewater can reduce adsorption performance [97]. In this article, GO coated with Fe_3_O_4_ is studied for its ability to adsorb pharmaceuticals from water, an application of increasing environmental importance. The research highlights the impact of NOM on adsorption efficiency and explores how tuning the pore dimensions of GO-Fe_3_O_4_ can enhance selectivity. This work is significant for the development of adsorbent materials capable of removing pharmaceuticals from wastewater, addressing a critical environmental challenge.

*A Facile Fabrication of Ordered Mesoporous Carbons Derived from Phenolic Resin and Mesophase Pitch via a Self-Assembly Method (Contribution 10) [98]*

Supercapacitors, also known as electric double-layer capacitors (EDLCs), are gaining attention as sustainable energy storage devices due to their high-power density, long cycle life, and fast charge/discharge capabilities [99,100,101]. Activated carbon is commonly used as the electrode material because of its large surface area and low cost. However, its low conductivity and microporous structure limit performance [102,103]. Ordered mesoporous carbon (OMC) offers better conductivity and tunable pore structures, making it more suitable for EDLCs [104,105]. They can be synthesized through hard- and soft-template methods, with the latter being more flexible and cost-effective. Mesophase pitches have been used to create high-quality mesoporous carbon by self-assembly processes, leading to improved electrochemical properties in supercapacitors [105,106]. This paper presents a method for fabricating ordered mesoporous carbons with high surface areas and tunable pore structures. The materials are synthesized using a self-assembly method and exhibit promising electrochemical properties, making them suitable for applications such as supercapacitors. The ability to control the mesostructure of these carbons opens up new possibilities for their use in energy storage devices, demonstrating the versatility of carbon-based nanomaterials.

**Table 1:** Overview of the articles with the key aspects of each study on porous nanomaterials, including nanomaterial type, scientific focus, and application.

Contribution Number	Porous Nanomaterial	Scientific Focus	Application
1 [1]	Silica nanoparticles	Stability of silica sol slurries for chemical mechanical polishing	Silicon wafer polishing in integrated circuit fabrication
2 [11]	Mesoporous silica nanoparticles (MSNs)	Synthesis and crystallization behavior of ultra-small templated mesoporous silica nanoparticles	Catalysis and separation support
3 [22]	Magnetic mesoporous silica nanoparticles	Gram-scale synthesis, functionalization, and nanosafety evaluation	Metal ion separation
4 [30]	Metal–Organic Framework (UiO-66-NH_2_)	Synthesis and characterization of UiO-66-NH_2_ composite for phosphate adsorption	Phosphate removal from wastewater
5 [48]	Metal–Organic Frameworks (MOFs)	Comparative adsorption of hydrocarbons (benzene, cyclohexane, n-hexane) on various MOFs	Hydrocarbon separation and adsorption
6 [59]	Metal–Organic Frameworks (MOFs)	Computational screening of MOFs for ethylene purification from complex mixtures	Ethylene purification
7 [2]	Zeolite (TS-1)	Influence of TS-1 on the electrical and thermal properties of crosslinked polyethylene composites	High-voltage direct current transmission systems
8 [81]	Zeolite (13X, FAU topology)	Study of the adsorption behavior of water, methanol, and cyclohexane on 13X zeolite	Selective adsorption in vapor-phase mixtures
9 [87]	Graphene oxide coated with magnetite (GO-Fe_3_O_4_)	Pharmaceutical adsorption influenced by natural organic matter and pore dimension tuning	Pharmaceutical wastewater treatment
10 [98]	Ordered mesoporous carbons (OMCs)	Synthesis of ordered and disordered mesoporous carbons and their electrochemical properties	Energy storage devices (supercapacitors)

The articles in this Special Issue highlight the diverse and rapidly evolving field of porous nanomaterials, demonstrating their broad applicability across various domains. From improving semiconductor manufacturing processes to addressing environmental pollution and enhancing energy storage systems, these materials offer significant potential for innovation. The advancements in the synthesis, characterization, and application of silica nanoparticles, MOFs, zeolites, and carbon-based nanomaterials showcased in this issue underscore the importance of continued research in this area. As the understanding of porous nanomaterials deepens, so too will their impact on industries ranging from electronics to environmental science and energy technology.
